# Making it hard to replicate

**DOI:** 10.7554/eLife.88044

**Published:** 2023-04-28

**Authors:** Billy Wai-Lung Ng, Stephan Scheeff, Josefina Xeque Amada

**Affiliations:** 1 The Chinese University of Hong Kong Hong Kong Hong Kong

**Keywords:** biomolecular condensates, influenza A virus, proteome-wide solubility, viral inclusions, phase transitions, viral condensates, Human, Mouse, Viruses

## Abstract

Understanding how to harden liquid condensates produced by influenza A virus could accelerate the development of novel antiviral drugs.

**Related research article** Etibor TA, Vale-Costa S, Sridharan S, Brás D, Becher I, Mello VH, Ferreira F, Alenquer M, Savitski MM, Amorim MJ. 2023. Defining basic rules for hardening influenza A virus liquid condensates. *eLife* **12**:e85182. doi: 10.7554/eLife.85182.

Understanding how viruses infect cells, replicate, and subsequently spread through the body is crucial for developing effective antiviral therapies. During this process, most common viruses – including the one responsible for flu, influenza A virus – form membrane-less organelles called condensates which help the virus to assemble its genome and replicate (Li et al., 2022). While some small molecules can manipulate the properties of these condensates to prevent viruses from replicating (Risso-Ballester et al., 2021), more research is required to understand how to efficiently and specifically target selected condensates.

Influenza A virus is thought to induce condensates in order to help with genome assembly (Alenquer et al., 2019). Its genome comprises of eight RNA segments, each forming a viral ribonucleoprotein (vRNP) complex that is synthesized in the nucleus. Once formed, the vRNPs migrate to the cytosol where, with a host factor called Rab11a, they create condensates known as viral inclusions, which possess liquid-like properties (Noda and Kawaoka, 2010; Han et al., 2021). Now, in eLife, Maria João Amorim and colleagues – including Temitope Akhigbe Etibor as first author – report how the material properties of these viral inclusions are maintained and regulated in live cells infected with influenza A virus (Etibor et al., 2023).

The team (who are based at Instituto Gulbenkian de Ciência, European Molecular Biology Laboratory and Católica Biomedical Research Centre) monitored the structure and orientation of viral inclusions by measuring their number, shape, size, and density. How the inclusions moved and interacted with each other was also studied through live-cell imaging and by calculating their molecular stability (Banani et al., 2017). Etibor et al. then investigated the impact of different factors on the material properties of the viral inclusions, including temperature, the concentration of vRNPs and Rab11a, and the number and strength of interactions between vRNPs (Figure 1). This allowed them to determine which of these factors has the greatest effect, and how these pathways may be manipulated to develop a new antiviral approach.

**Figure 1.**
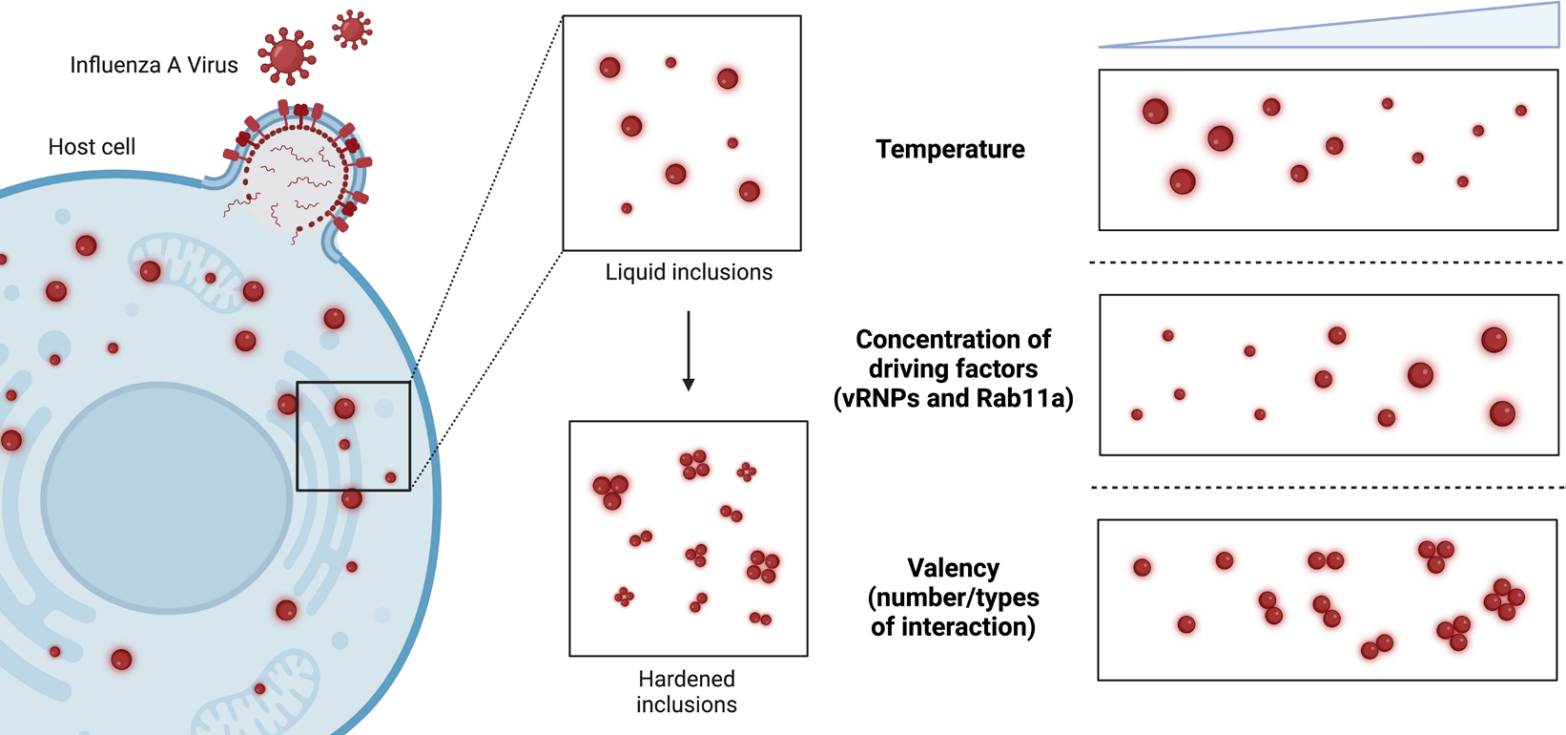
Modulating the material properties of the viral inclusions formed by influenza A virus. During infection, influenza A virus (red) enters host cells (blue) and replicates. To achieve this, two driving factors – viral ribonucleoproteins (vRNPs) and a protein called Rab11a – drive the formation of liquid condensates called viral inclusions (top middle inset). It is thought that hardening these viral inclusions (bottom middle inset) so that they become stiffer and less round will make it more difficult for viruses to replicate and assemble their genomes. Etibor et al. investigated how changes in temperature, the concentration of the driving factors, and the valency (i.e., the number/types of interactions among the vRNPs) affected the properties of influenza A virus inclusions. Raising the temperature and concentration of driving factors led to smaller and larger viral inclusions respectively, but had no effect on the material properties of the viral inclusions (top and middle right inset). Increasing the valency led to more rigid viral inclusions, which were unable to fuse together and lost many of their liquid characteristics (bottom right inset).

Image credit: Figure created with BioRender.

The experiments revealed that while changes in temperature and the concentration of vRNPs and Rab11a altered the size of the viral inclusions, the material properties of the inclusions remained mostly the same. These results are surprising as previous studies have shown that, in general, condensates strongly depend on these two factors. Etibor et al. noted that these findings may be specific to influenza A virus, as its condensates need to maintain liquid-like properties over a wide range of vRNP concentrations to replicate efficiently.

Next, Etibor et al. treated cells with nucleozin, a pharmacological modulator that has been shown to lower the viral load in patients with influenza A in preclinical studies (Kao et al., 2010). Nucleozin glues together nucleoproteins (the major components of vRNPs), expanding the number and type of interactions within individual vRNPs as well as between different complexes. The increased interactions stabilized the vRNPs and led to more rigid and less dynamic viral inclusions which did not dissolve following shock treatments and were less able to fuse together. This suggests that nucleozin hardens the material properties of viral inclusions by increasing interactions between vRNPs.

The team also showed that nucleozin stiffened viral inclusions in the lung cells of mice infected with influenza A virus, and helped speed up the mice’s recovery. Furthermore, nucleozin did not alter the level of other proteins in the cells of the mice, demonstrating the drug’s specificity against the virus.

In summary, Etibor et al. revealed how different factors influence the material properties of viral inclusions in both cells and mice infected with influenza A virus. Their findings suggest that stabilizing vRNP interactions shows the most promise for disrupting the function of viral inclusions, and highlight the potential of antiviral drugs that harden these condensates.
